# Protein Nucleation and Crystallization Process with Process Analytical Technologies in a Batch Crystallizer

**DOI:** 10.1021/acs.cgd.3c00411

**Published:** 2023-06-20

**Authors:** Wenqing Tian, Wei Li, Huaiyu Yang

**Affiliations:** Department of Chemical Engineering, Loughborough University, Loughborough LE113TU, U.K.

## Abstract

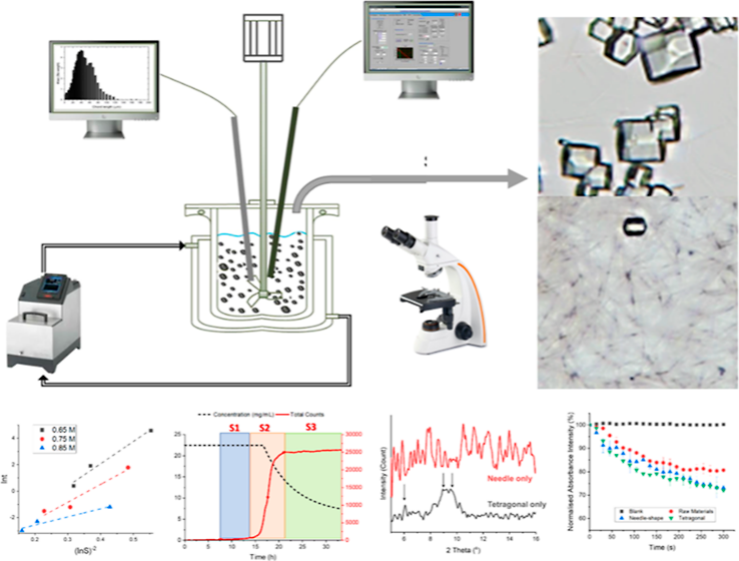

Protein crystallization has drawn great attention to replacing the traditional downstream processing for protein-based pharmaceuticals due to its advantages in stability, storage, and delivery. Limited understanding of the protein crystallization processes requires essential information based on real-time tracking during the crystallization process. A batch crystallizer of 100 mL fitted with a focused beam reflectance measurement (FBRM) probe and a thermocouple was designed for in situ monitoring of the protein crystallization process, with simutaneously record of off-line concentrations and crystal images. Three stages in the protein batch crystallization process were identified: long-period slow nucleation, rapid crystallization, and slow growth and breakage. The induction time was estimated by FBRM, i.e., increasing numbers of particles in the solution, which could be half of the time required for detecting the decrease of the concentration, by offline measurement. The induction time decreased with an increase in supersaturation within the same salt concentration. The interfacial energy for nucleation was analyzed based on each experimental group with equal salt concentration and different concentrations of lysozyme. The interfacial energy reduced with an increase in salt concentration in the solution. The yield of the experiments was significantly affected by the protein and salt concentrations and could achieve up to 99% yield with a 26.5 μm median crystal size upon stabilized concentration readings.

## Abstract 2

Three stages with different nucleation and concentration changes were identified. It was the very slow kinetics that led to hours delay of the secondary nucleation after the occurrence of first nucleation. The needle-shaped crystals appeared in some conditions but always after the tetragonal crystals. The high bioavailabilities of these crystals show that crystallization can be a better method to maintain high bioavailability than lyophilization.

## Introduction

Bioengineered protein-based drugs have attracted significant commercial interest and have become one of the fastest expanding pharmaceuticals worldwide. The global market is expected to grow at a rate of 7.4% annually, reaching a market value of $337 billion by 2025.^1^ To satisfy the growing demand, protein crystallization is considered as a potential technology for protein purification, with products of higher stability and greater purity, and is less costly and easy to scale up compared to the current preparative chromatography methods.^2,3^

The protein crystallization methods have been developed at the laboratory level, but there are challenges in the scaling-up process.^2^ The challenges include difficulties in controlling the nucleation due to the lack of accurate information of protein nucleation kinetics.^3,4^ It is difficult to estimate the supersaturation during the crystallization in the microscale experiments, such as the widely used vapor diffusion experiments, and it is not possible to estimate the nucleation thermodynamic parameters. High-throughput crystallization experiments for protein crystals could identify the suitable nucleation conditions but cannot provide the mixing conditions for the kinetic parameters for the scaling-up process.^5^ Some attempts have been tested on larger scales with a stirred tank, which is commonly used in crystallization of small molecules.^6^ Judge et al. conducted ovalbumin purification to a purity greater than 99% in a 1 L batch crystallizer.^7^ Isoforms of a new protein-active ingredient were able to be separated in 100 mL scale stirred tanks.^8^ The crystallizer with lower shear forces with less energy input was found to perform better than the one with larger forces and higher energy input. The recombinant l-methionine γ-lyase, an anticancer agent, was crystallized with an 87% yield in a 100 L crystallizer.^9^ The formation of two different forms of lysozyme was discovered in the batch crystallizer: needle-shaped crystals formed in low lysozyme concentration and tetragonal-like crystals formed in high concentration.^10^ Different modes of operation, batch, continuous, and airlift, were also found to affect the morphology of protein crystals. Nucleation times of 80 h in 3.0% (w/v) NaCl, 28 h in 3.5% (w/v) NaCl, and 4 h in 4.0% (w/v) NaCl concentration were achieved in the stirred tank crystallizer.^11^ There is limited research on the scaling up of protein crystallization with little information reported on the thermodynamics and kinetics of the protein crystallization process at different scales.

Process analytical technology (PAT) tools can be applied to observe real-time solute concentration, crystal counts, and crystal size distribution (CSD).^12,13^ Focused beam reflectance measurement (FBRM), Coulter counter, laser diffraction, ultrasonic attenuation spectroscopy, and microscope image analysis were employed in tracking the crystal size change in the crystallization process.^14−16^ The rotating laser beam is able to record the counts and the chord length distribution (CLD) using its intrinsic algorithm, and the CLD will be translated to CSD. In recent years, the methods for translating from CLD to CSD in the crystallization process have included the spherical equivalent diameter (SED) model^16^ and the crystal size database based on experimental works.^17,18^ It is common to couple FBRM with particle vision and measurement (PVM) to visualize the crystallization process and calibrate the CSD in the system.^13,19^ UV/vis and IR spectroscopies using a distinctive spectrum of a chemical compound and the characteristic wavelength peak can be used to detect the concentrations of different compounds in the solution^20^ based on Beer–Lambert law.^21^ Until now, very few research on the protein crystallization process with PAT tools hinders the understanding of the crystallization process for scaling up and optimizing the protein crystallization process.

In this work, the crystal shape, size distribution, and concentration were first-time systemically and simultaneously recorded in protein nucleation and crystallization processes, by FBRM, off-line measurements of a spectrophotometer and an optical microscope, as well as PVM and UV/vis spectroscopy. The crystallization experiments were performed in a 100 mL stirred tank batch crystallizer with lysozyme concentrations from 15 to 35 mg/mL and a NaCl concentration of 0.65–0.85 M. Three stages during the crystallization processes were identified based on the changes of concentration and CSD. The interfacial energy of lysozyme in the solution was estimated by determination of the induction time. The yield and size of the crystal product were characterized and analyzed.

## Experimental Section

### Materials

Hen egg white lysozyme (∼70,000 units/mg protein), sodium acetate (purity >99%), sodium chloride (purity >99.5%), and glacial acetic acid (purity >99.5%) were purchased from Sigma-Aldrich and used without further purification. Reverse osmosis (RO) water was prepared with the RO water purification system (Merck Millipore, Synergy UV) with 12.3 MΩ cm electrical resistivity and 20 °C.

### Crystallization of Lysozyme

The buffer solution was prepared as 0.1 M sodium acetate in RO water at pH 4.2 ± 0.002 by addition of acetic acid using a pH meter (SciQuip Precision benchtop pH meter). Lysozyme solutions with concentrations of 30, 50, and 70 mg/mL were prepared by dissolving lysozyme in sodium acetate buffer solution, and NaCl solutions were prepared using the same method to achieve concentrations of 1.3, 1.5, and 1.7 M. All solutions were filtered through a 0.2 μm cellulose acetate filter and stored at 20 ±0.2 °C in the incubator (VWR INCU-Line 150R) before experiments. The lysozyme solution and NaCl solution were mixed with 1:1 ratio in the 100 mL stirred tank crystallizer. A three-blade marine-type propeller with an impeller to a vessel diameter of 0.5 with a speed of 200 rpm (Heidolph RZR2021 motor, Heidolph Instruments) was deployed to achieve homogeneous mixing in the solution.^22,23^

**Figure 1:**
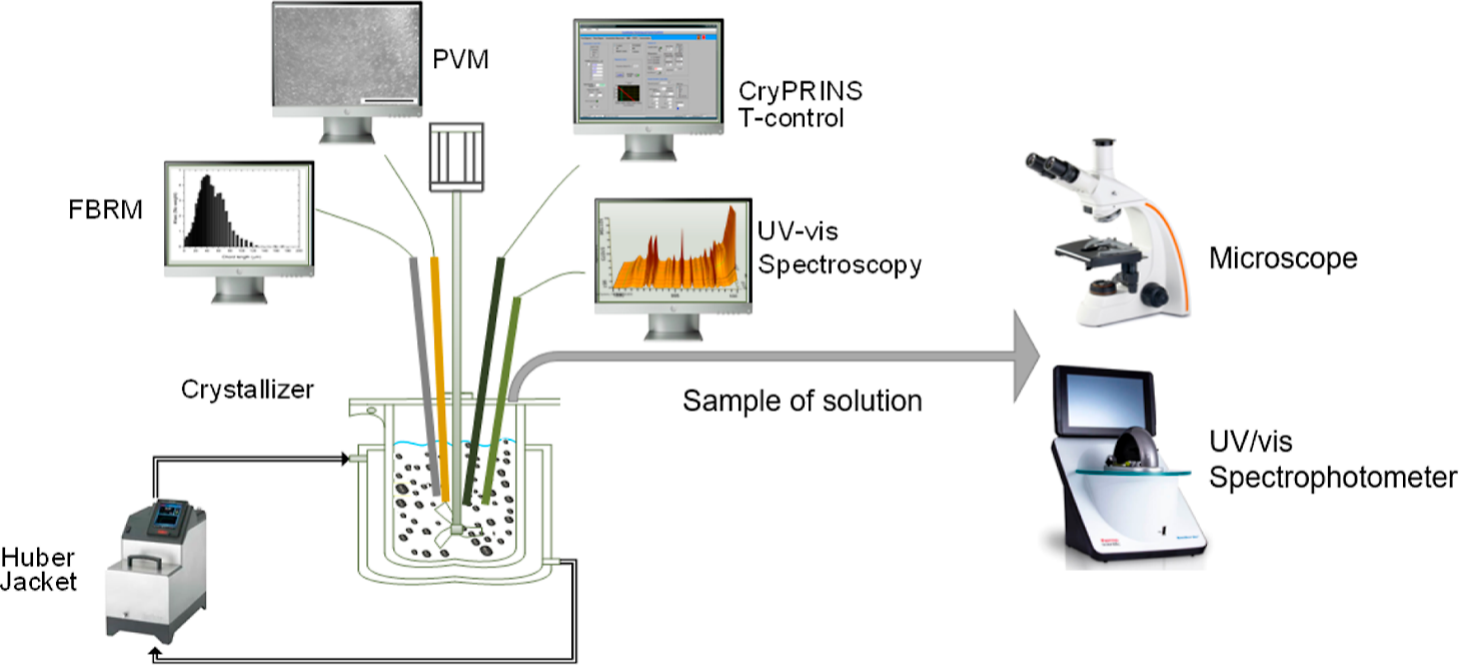
Schematic representation of the experimental setup.

As shown in Figure 1, an FBRM probe (model G400, Mettler Toledo) was immersed in the solution at a fixed position to measure the particle counts and CLDs of protein crystals in the real time. The induction time was estimated by the first obvious change of the total particle count based on the FBRM data. The measurement duration was set as 5 s. A V819 PVM probe (Mettler Toledo PVM) was applied to visualize the crystallization process in situ with a frame rate of 10 Hz. The in situ protein concentration was measured by a calibrated spectroscopy probe, MSC621 Carl Zeiss ATR-UV/vis probe, with a software (LabView) to collect UV/vis spectra. The temperature of the crystallizer was kept constant at 20 °C by a thermostat water bath (Ministat 125 with Pilot ONE, Huber) and a thermocouple fitted with the crystallization process informatics system (CryPRINS) software. CryPRINS software was used to record the process with a variety of process analytical technology tools.

Conditions of various lysozyme concentrations of 15–35 mg/mL and NaCl concentrations of 0.65–0.85 M after mixing were used in crystallization, as shown in Table 1. The induction time and other nucleation parameters were examined for all experiments. The crystal size and yield of exps 1–3, 5, and 8 were compared at 24 h and at the final stage of the experiment, when there was less 10% of protein concentration change in 5 h. Samples of 0.2–0.3 mL of solutions were extracted from the bulk crystallization solution every 30 min and observed under a microscope (GT Vision GTC-20). The protein concentrations of the sample solutions were determined by a UV/vis spectrophotometer (Thermo Scientific Nanodrop One) at 280 nm after centrifugation (SciSpin One Compact Centrifuge) for 3 min at 15.0 g. The determination of protein concentration typically uses 280 nm as the characteristic peak due to the strong light absorption of aromatic amino acids tryptophan (Trp) and tyrosine (Tyr).^24^ The concentration of lysozyme can be calculated with an extinction coefficient of 2.64 L/g for lysozymes and the absorbance at 280 nm.^25^ Each measurement was repeated at least three times.

**Table 1:** Lysozyme Crystallization Experimental Conditions at 20 °C with a pH of 4.2 and Estimated Nucleation Parameters

experiment number	*C*_NaCl_ (M)^a^	*C*_Lys_ (mg/mL)	*S*	*t* (h)	ln *A* [ln (s^–1^ m^–3^)]	σ (mJ/m^2^)	*G* (kJ/mol)
exp 1	0.65	15	3.4	98.2	9.39	0.423	34.6
exp 2^b^		25	5.3	6.8			17.4
exp 3		35	7.4	1.5			11.8
exp 4	0.75	15	4.0	6.0	4.81	0.393	18.1
exp 5		25	6.3	0.3			10.2
exp 6		35	9.2	0.2			7.6
exp 7	0.85	15	5.4	0.3	3.83	0.302	6.0
exp 8		25	9.3	0.1			3.2
exp 9		35	12.9	0.05			2.4

a *C*_NaCl_: concentration of NaCl after mixing; *C*_Lys_: concentration of lysozyme after mixing; *S*: supersaturation ratio; *t*: induction time; *A*: pre-exponential factor; σ: interfacial energy; *G*: critical nucleation free energy.

b The same condition for three different runs.

### Solubility Measurement

At the end of the experiments, the supernatant solution from the batch crystallization experiments was stored at 20 °C until equilibrium (up to 3 months). The supernatant solution after centrifugation was measured by a spectrophotometer for determining the lysozyme concentration. The lysozyme concentration at equilibrium was estimated as the lysozyme solubility under the experimental condition. Each measurement was repeated at least three times.

### Size of Crystals

Four different methods were employed in examining the CSD in the lysozyme batch crystallization process: (1) FBRM converted the CSD based on the SED model, (2) converted the CSD from CLDInversion software,^17,18^ (3) microscopic image analysis, and (4) laser diffraction measurements from a Mastersizer (Malvern Mastersizer 2000, Malvern Instruments, UK). All crystal sizes were taken as number average.

For the first method, the CLD by FBRM was converted into crystal size based on the SED model.^26^ The probability (*P*) of measuring the chord length between two specific lengths, *l*_1_ and *l*_2_ (unit in μm), on a sphere with a diameter of *D* (unit in μm) was calculated as
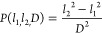
1

CLDInversion software was used to compute the global best solution fitted for non-spherical crystals by using a large amount of crystal size data database^17,18^ and was used as the second method for finding the CSD for protein crystals.

The third method was to observe the sample solution under an optical microscope. The microscopic images of the crystals were analyzed with ImageJ software (Wayne Rasband, National Institute of Health, USA) to calculate the CSDs. The area, *A* (unit in μm^2^), was measured by automatically calculating using the software to enclose the crystal edges in the images, which was then converted into crystal size. The crystal size was estimated by using the circular equivalent diameter, *D* (unit in μm),^27^
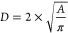
2

For the fourth method, the crystal slurry at the end of the experiment was transferred into a funnel and vacuum-filtered by filter paper with 0.22 μm pore size (GVWP filter, Millipore Corp., MA, USA). The crystals were then dried at 20 °C in a drier until the constant weight was achieved. The sample was then inserted into a laser diffractor (Malvern Mastersizer 2000) for particle size analysis. Isopropyl alcohol (IPA) was selected as the medium to disperse lysozyme crystals in the laser diffractor due to lysozyme’s low solubility in IPA, and the refractive index was selected to be 1.4.

### X-ray Powder Diffraction

Filtered and dried crystals were subjected to X-ray powder diffraction (XRD) on a Bruker D8 Discover diffractometer (Bruker AXS GmbH) at 20 °C with a Cu Kα source. The diffraction data were collected under 40 kV and 40 mA from 3 to 20° (2θ) at a stepwise of 0.1°.

### Activity of the Protein

The biological activity of the produced lysozyme crystals was estimated by using a lysozyme activity kit (Sigma-Aldrich). Micrococcus lysodeikticus cells were employed as the substrate with a concentration of 0.01% (w/v), and active lysozyme resulted in the lysis of the cells. The same amount of lysozyme crystal products and the reference product in the activity kit (≥40,000 active units/mg) were diluted with the reaction buffer and mixed with *Micrococcus lysodeikticus* cell suspension. The concentration of cells was monitored by Nanodrop One at 450 nm for 5 min at a constant room temperature.

## Results

### Induction Time

The solubility of lysozyme in 0.1 M acetate buffer (pH = 4.2) at 20 °C decreased with the increase of NaCl concentration, as shown in Figure S1. The solubility was consistent with the solubility of tetragonal lysozyme crystals reported.^28^ Supersaturation, *S*, can be calculated by the ratio of the solution concentration in mg/mL, *c*, and the solubility, c*, as
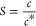
3and the supersaturation for various protein and salt concentrations, listed in Table 1, was estimated based on the solubility curves shown in Figure S1.

**Figure 2:**
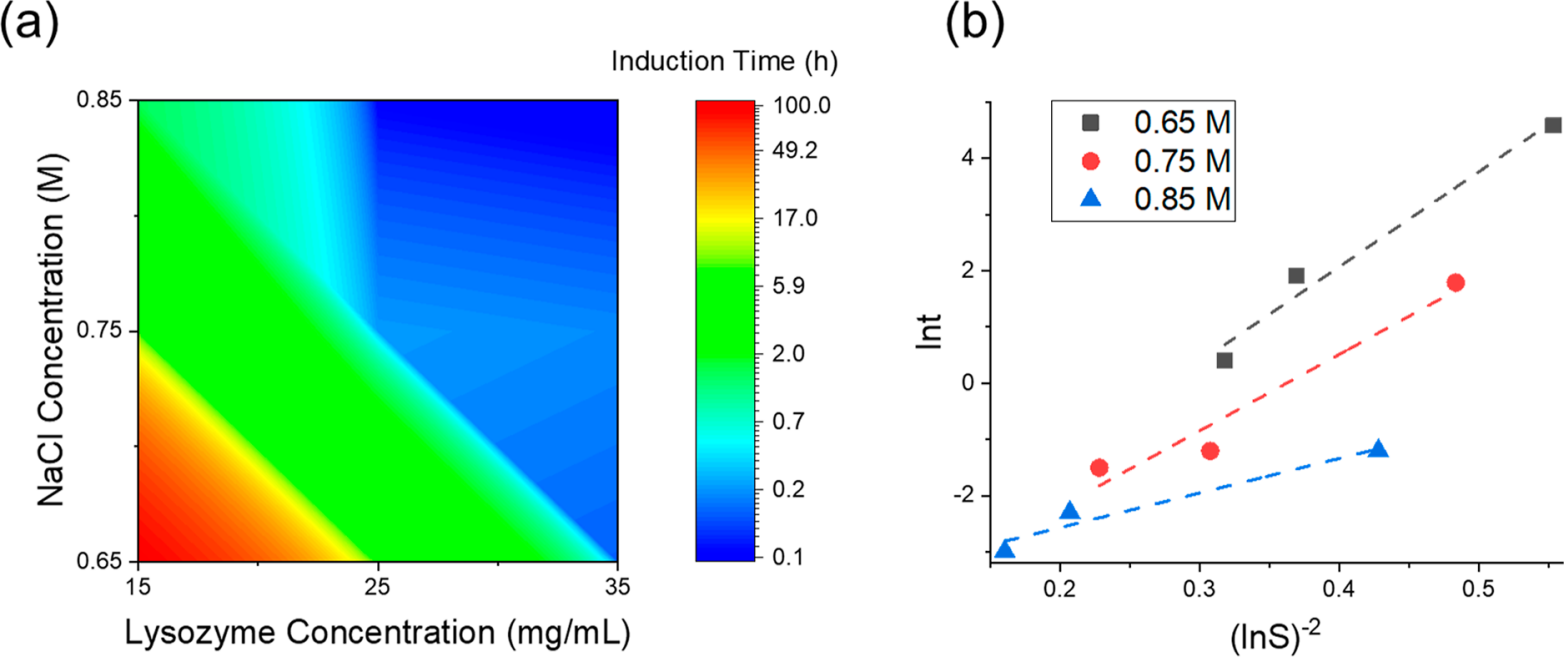
Effect of lysozyme concentrations, NaCl concentrations, and supersaturation on the induction time of the batch crystallization process. (a) Induction times in the solutions with different concentrations of lysozyme and NaCl. (b) Correlations between supersaturations and induction times with unit of h. Dashed lines are the best-fitting line of ln *t* in hours against (ln *S*)^−2^ at 0.65, 0.75, and 0.85 M NaCl, respectively.

The induction time decreased with the increase in lysozyme or NaCl concentrations, shown in Figure 2a, whose trend was consistent with the results of microscale experiments.^27^ The induction time in the crystallization process is defined as the time between the establishment of the supersaturation and the occurrence of the nucleation,^29^ which was often approximated as the observation of the first crystal in the solution.^30^ In this work, FBRM was used to detect the onset of crystal count increase as the starting point of the nucleation. With 0.65 M NaCl, the longest induction time was 98.2 h in the solutions with 15 mg/mL lysozyme. The induction time significantly reduced to 0.2 h in the solution with 35 mg/mL lysozyme. With 15 mg/mL lysozyme, the induction time reduced to 0.3 h in the solution with 0.85 M NaCl from 98.2 h in the solution with 0.65 M NaCl. With 25 mg/mL lysozyme, the induction time with 0.65 M NaCl was the longest, 6.8 h, and reduced to 0.1 h when the NaCl concentration increased to 0.85 M. Exp 2 with 25 mg/mL lysozyme and 0.65 M NaCl was repeated three times, with a standard deviation of 14% for the induction time, showing a good consistency with the trend. The fastest nucleation occurred at 0.85 M NaCl and 35 mg/mL lysozyme condition, which only required 0.05 h to detect the start of nucleation.

Based on the classical nucleation theory, the induction time, *t* (unit in s), is a variable of supersaturation, *S*, and temperature, *T*, with the unit of K^29,31^
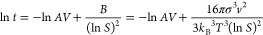
4where *A* is the pre-exponential factor with the unit of s^–1^ m^–3^, which is considered to be constant at different supersaturations,^29^*V* is the solution volume with the unit of m^3^, *B* is the slope of the fitting lines in Figure 2, σ is the interfacial energy in critical cluster formation with the unit of mJ/m^2^, *v* is the molecular volume of lysozyme crystal with the unit of m^–3^, and *k*_B_ is the Boltzmann constant with the unit of J/K. With 0.65–0.85 M NaCl concentrations, three linear correlations between ln *t* vs (ln *S*)^−2^ are shown in Figure 2b. As the induction time was denoted in hours instead of seconds in SI units, eq 4 was subtracted by 8.19 to adjust for the unit change. It is noted that two-step nucleation was reported in protein crystallization systems,^32^ but for the estimation of the nucleation parameters to compare the difficulty of the nucleation process under different conditions, homogeneous nucleation was assumed in this work with applying classical nucleation theory.

The slope, *B*, of the straight lines decreased from 16.9 at 0.65 M to 6.2 at 0.85 M NaCl concentration. The higher the slope was, the more difficult the nucleation became. The energy barrier required for the formation of the lysozyme critical nuclei decreased with an increase in NaCl concentrations in the bulk solution. It was reported that NaCl reduced the solvation layer around the protein molecules and promoted isolated lysozyme molecules gathering for nucleation.^2,33^ The interfacial energy, σ, in critical nuclei formation was determined from the slope, *B*, and is summarized in Table 1. For different experimental conditions with a molecular volume of lysozyme 2.97 × 10^–26^ m^3^.^34^ At 20 °C with 0.65 M NaCl, the interfacial energy was estimated as 0.423 and 0.302 mJ/m^2^ with 0.85 M NaCl. The interfacial energy of lysozyme was reported as 0.1–0.3 mJ/m^2^ for 0.5 to 0.85 M NaCl at pH 4.5 buffered solution,^35^ which was in the similar range as the values in this work. The critical free energy decreased with the increase of the supersaturation under the same NaCl concentration, as shown in Table 1, which was in the range of 2–35 kJ/mol. Correspondingly, the critical diameters of the nuclei were estimated to be in the range of about 4–8 nm. The pre-exponential factor, *A*, can be determined by the interception of the linear line on the *y*-axis, shown in Figure 2b, by eq 4. With the increase in NaCl concentration in the solution, ln *A* decreased in the range between 9.39 and 3.83, as shown in Table 1. In summary, there was no overall trend based only on the supersaturation level, and besides the supersaturation, the NaCl concentration plays a very important role in the nucleation of lysozyme.

### Crystallization Process

**Figure 3:**
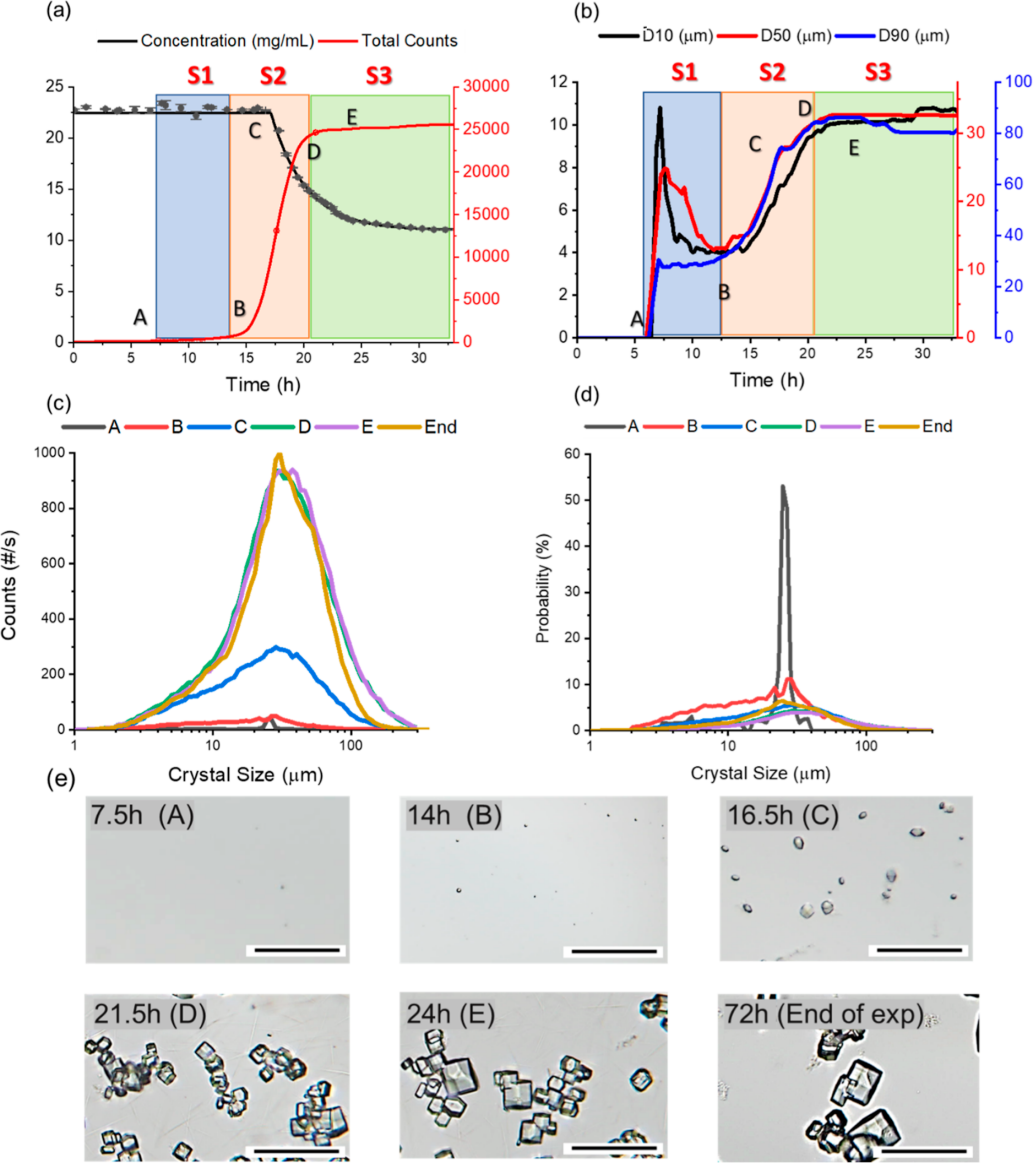
Change of lysozyme concentration and total counts (a); D10, D50, and D90 (b); CSDs in counts (c), size distribution in probability (d), and crystal shape by microscopic images (e) with a scale bar of 100 μm during the crystallization process of exp 2.

Figure 3 shows the change of concentrations, particle counts, and crystal sizes during the crystallization process of exp 2. The nucleation was detected at point A, the start of the FBRM curve, and the nucleation time was about 6.0 h under about a supersaturation of 5.2. After nucleation (point A), there were three stages: a long-period slow nucleation stage (stage 1), a rapid nucleation and growth stage (stage 2), and a slow growth and breakage stage (stage 3), highlighted with blue, orange, and green regions in Figure 3a, respectively.

During stage 1 (point A to B), at point A, no crystal in the sample solution was observed under the microscope. The nucleation started at point A where the total counts started to increase. The particle numbers increased slowly during this stage, and the appearance of crystals was supported by the change of D10, D50, and D90 in Figure 3b. Figure 3c,d show that, at the time close to point A, the crystal number in the solution was low, and there was a narrow distribution with a D10 of 11.1 μm and a D90 of 20.8 μm. The decrease of D10 and D50 after a maximum value in Figure 3b revealed the appearance of many tiny lysozyme crystals in the system, indicating that the primary nucleation dominated in this stage due to the high level of supersaturation.^36^ However, there was no burst of crystal number as in most of the nucleation processes of small molecules,^2,8,37^ as the total counts slowly increased for only 600 in a period of more than 10 h. The size distribution at point B became much wider compared to that at point A, and the large probability of increase was small crystals, as shown in Figure 3d. The total counts were still under 800 at point B.

At stage 2 (point B to D), the total number of crystals increased rapidly with a fast growth in the average size of crystals, as shown in Figure 3a,b. Some tiny crystals were observed under the microscope at point B, shown in Figure 3e, which was consistent with the FBRM curves, showing a very low crystal density and small median size in Figure 3a,b. The D50 of the crystals had the lowest value at around point B; then, the average crystal size became larger. The large number of crystals quickly appeared after point B, and the increase rate of crystal numbers was about 290 times of that in stage 1. The total counts reached above 10,000 at point C with a mean crystal size of 23.2 μm. A wider CSD at point C with higher crystal counts than that at point B was observed, as shown in Figure 3c,d. After point C, the concentration started to decrease, triggered by the fast nucleation and crystal growth. The supersaturation decreased with the decrease in the concentration, and only half of the supersaturation left as the initial point in about 6 h. The solution sample under the microscope also showed an increase in the number of crystals and an increase in average size, as demonstrated in the images of points B, C, and D in Figure 3e.

It is surprising to observe that there was no obvious change in the concentration for the long period after nucleation from point A to C (during stage 1 and the first half of stage 2). The concentration remained at the initial value for more than 7 h even after the formation of hundreds of crystals (proved by FBRM and microscopic images). However, these crystals were all very tiny, and the number only slowly increased, limiting the influence on the concentration. For many of the small organic molecular crystallization systems,^2,8,37^ the concentration started to quickly drop as the start of the nucleation with the formation of a huge number of crystals, and the concentration decrease can be used as an indicator of the nucleation. However, in exp 2, the change of concentration occurred at nearly double the time longer than the start of the nucleation. FBRM captured a much earlier sign of nucleation at point B (7.5 h) than the drop of the concentration at point C (16.5 h). There were similar phenomena in other experiments, exps 1–3, 5, and 7, with an average of 3.9 h gap between the nucleation and the time when the concentration started to drop.

The microscopic images of the sample solution showed an earlier sign of nucleation by observing tiny crystals in the solution than the concentration change, and FBRM curves revealed the earliest time compared to the microscopic observation and the concentration change. Therefore, FBRM identified a more accurate nucleation time. For exp 8 (the supersaturation was about 9.0), the induction time was observed to be 0.1 h, and the gap to the concentration drop was merely 0.5 h, as shown in Figure 3. The gap increased to 34 h for exp 1 with an induction time of 98.2 h (the supersaturation was about 3.5). It is noted that there was a positive correlation between the time of nucleation identified by FBRM and with time required for the concentration to drop. The concentration changes in the protein crystallization were used as an indicator of the nucleation,^11,22,23^ which could provide the difference in the difficulty of the nucleation. However, the concentration change is highly delayed after the nucleation, and the induction time cannot be estimated by the concentration change, and, therefore, the interfacial energy that is determined based on the concentration change is not reliable. If the time when the concentration started to drop was applied to correlate the supersaturation by eq 4, shown in Figure S2, the nucleation parameters extrapolated resulted in large variations. The estimated interfacial energy based on the time when the concentration started to drop had an average of 15% lower than those based on the induction time determined by FBRM.

At stage 3 (point D to end of experiment), the protein concentration continued to decrease, but the decrease rate was slower than the one in stage 2. The fraction of smaller lysozyme crystals decreased, and the percentage of crystals larger than 25.0 μm increased. The crystal number increased relatively fast until point D and started to stabilize (very slow increase) afterward. There were similar CSDs at point D and point E, and the peak slightly shifted to the right, proving that more large crystals existed at point E (D50 = 32.3 μm) than at point D (D50 = 31.4 μm). The decrease rate in the concentration after point E was slower compared with that before point E, which would lead to an increase in crystal size until the end of the experiment. However, the increase of the crystal number and the decrease of D90 in Figure 3b indicated that breakage had a stronger influence than the crystal growth after point E. The microscopic images in Figure 3c show an increase in the size of the tetragonal crystals from point D to point E, which was consistent with the size distribution and FBRM. At the end of the experiment (72 h), the smaller crystals grew into a slightly bigger size compared to point E, as supported by the reduced left tail of CSD in Figure 3c,d. The reduction of larger crystals in the CSD suggested a breakage of bigger crystals or agglomeration in stage 3.

**Figure 4:**
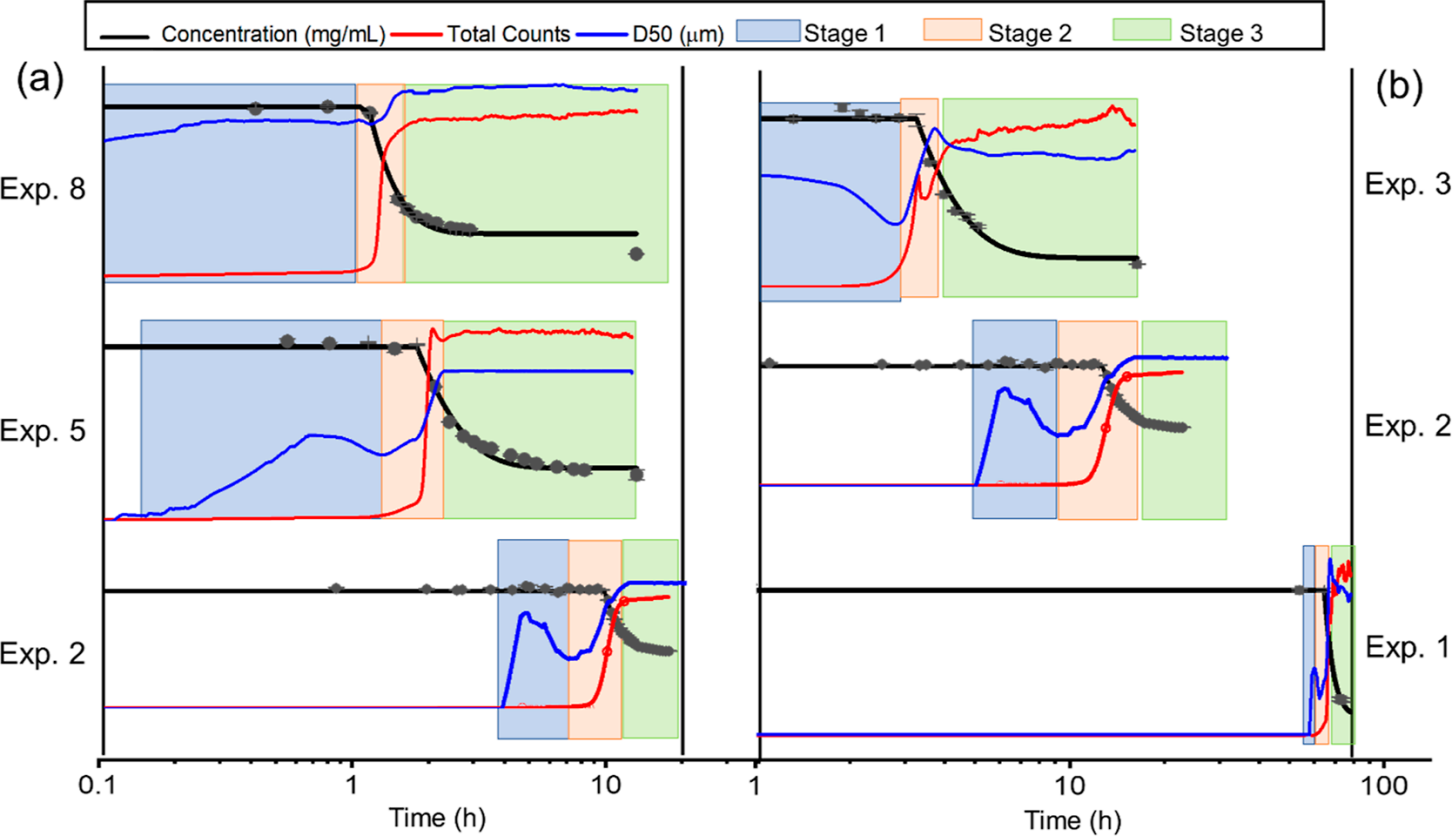
Change of lysozyme concentration, mean size, and total counts in three crystallization stages in the solutions with equal 25 mg/mL lysozyme and different NaCl concentrations (a) and with equal 0.65 M NaCl and different lysozyme concentrations (b).

The three crystallization stages were observed in all the experiments in this work. Figure 4a,b show the process in the solution with equal NaCl concentration and with equal protein concentration, respectively, and the supersaturation decreased from top to bottom for both figures. In exps 3 and 8, stage 1 nucleation started at 0.1 and 0.2 h, respectively. With increasing lysozyme concentration or NaCl concentration, the starting time of stage 1 became earlier, and the duration of stage 1 reduced. In solutions with equal 25 mg/mL lysozyme, the duration of stage 1 increased from 1.4 to 5.5 h when the NaCl concentration decreased from 0.85 to 0.65 M. In the solutions with 0.65 M NaCl, the duration of stage 1 increased from 2.9 to 8.3 h when the lysozyme concentration decreased from 35 to 15 mg/mL.

During stage 2, there were a large number of small crystals appearing in the solution. For stage 1, there were much fewer crystals, and D50 increased and decreased, reaching a low value at the beginning of stage 2. D50 at the beginning of stage 2 was in the range of 12.0 to 19.7 μm. The trend of D10 was similar to that of D50. D10 was about 2.5–4.5 μm at the beginning of stage 2, and D10 was 4.5 μm in the compared experiment with the highest supersaturation (exp 8). The relationship between the duration of stage 2 and concentrations of lysozyme or NaCl was similar to that of stage 1. The duration of stage 2 increased from 0.7 to 9.5 h when the NaCl concentration decreased from 0.85 to 0.65 M and increased from 1.2 to 10.6 h when the lysozyme concentration decreased from 35 to 15 mg/mL. The decrease in the protein concentration occurred in the middle range of stage 2, with the fast increase of the crystal numbers.

In stage 3, the increase in the total count was significantly lower than the one in stage 2 for all experiment sets. The concentration continued to decrease albeit at a slower rate. In the solutions of 0.65 M NaCl concentration, it was observed that D50 slightly decreased for all NaCl concentrations. The small increase in count and the decrease in D50 indicate that there was a breakage in stage 3. This observation was less obvious in solutions of 25 mg/mL lysozyme concentration, possibly due to the smaller crystals produced in these conditions. The duration of stage 3 was much longer than the one of stages 1 and 2, which suggests that the time to reach thermodynamic equilibrium in protein crystallization was very long after the major nucleation events happened. For solutions with equal 0.65 M NaCl concentration, the duration of stage 3 increased from 15 h in 35 mg/mL lysozyme solution to more than 200 h in 15 mg/mL lysozyme solution. With equal 25 mg/mL lysozyme in the solution, the duration of stage 3 increased from 10 h in solution with 0.85 M NaCl to 18 h in the solution with 0.65 M NaCl.

### Crystal Yield and Crystal Size

**Figure 5:**
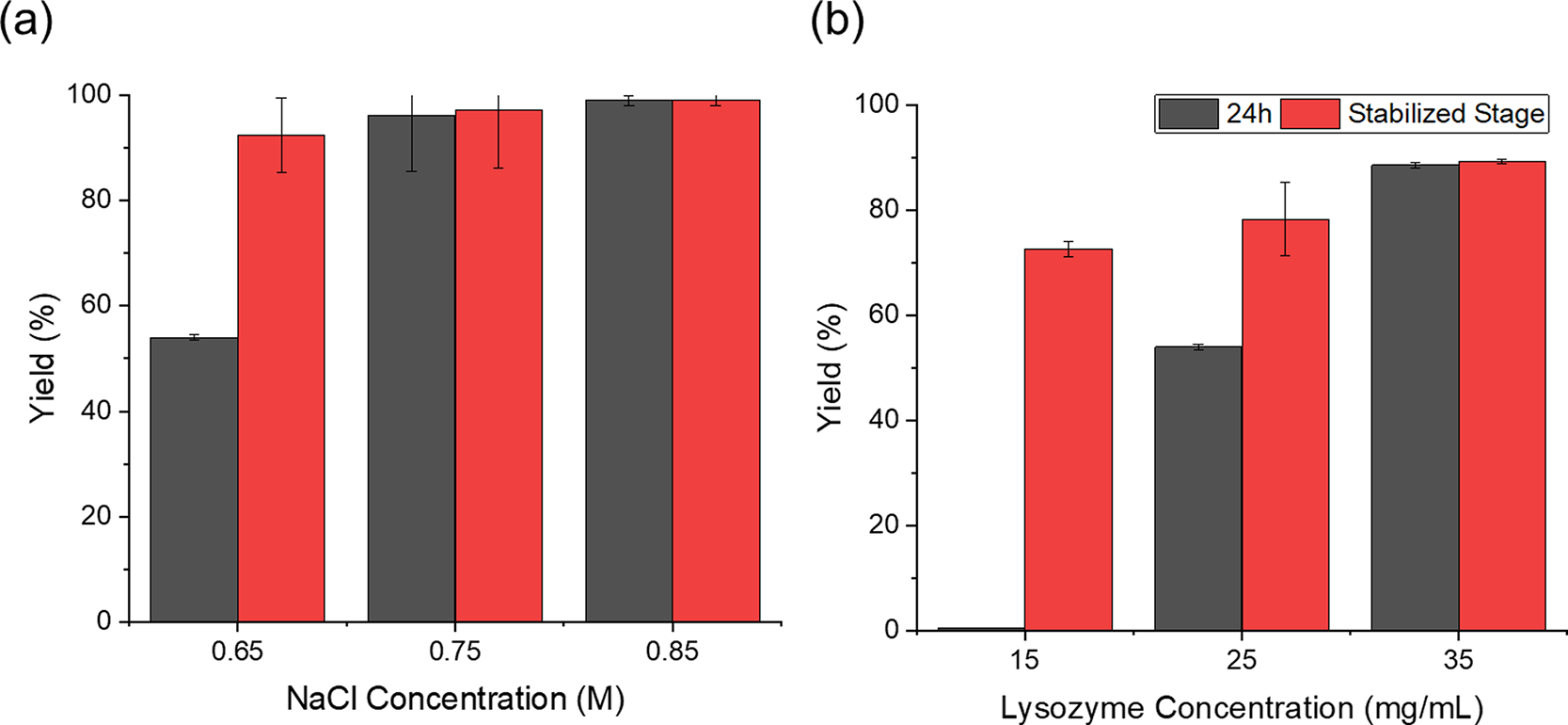
Protein crystal yield at 24 h and at the stabilized stage in the solutions with 25 mg/mL protein and different concentrations of NaCl (a) and 0.65 M NaCl and different concentrations of protein (b). Legend applies to both graphs.

**Figure 6:**
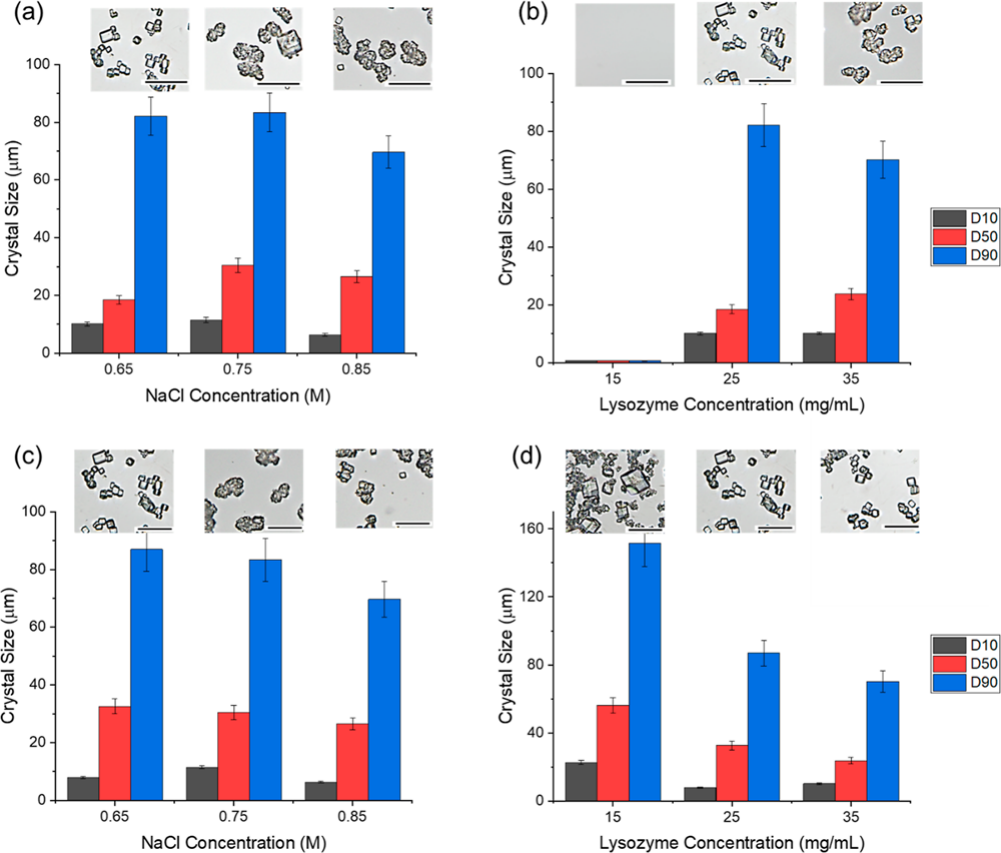
D10, D50, and D90 by FBRM and microscopic images of lysozyme crystals from the solutions with 25 mg/mL lysozyme and different NaCl concentrations at 24 h (a) and at the stabilized concentration (c) with 0.65 M NaCl and different lysozyme concentrations at 24 h (b) and at the stabilized concentration (d). Scale bars: 100 μm.

The protein crystal yield was estimated by the ratio of decreased protein concentration to the supersaturated concentration, the maximum amount of protein that can be crystallized out, and could be expressed as
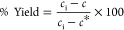
5where *c*_i_ is the initial protein concentration after mixing, *c* is the current protein concentration, and c* is the protein solubility with the unit of mg/mL. The yields at 24 h were compared for the equal concentration of protein or NaCl, as shown in Figure 5. The yields in the solution with 25 mg/mL lysozyme increased from over 50% to nearly 100% with the increase in the NaCl concentrations, shown in Figure 5a. The higher supersaturation led to faster nucleation and a correspondingly longer growth period and a larger crystal size. As shown in Figure 6a, the D50 of crystals was 18.5 μm with a condition of 0.65 M NaCl in the solution and was 30.4 μm with 0.75 M NaCl. A much higher nucleation rate would induce much more nuclei and crystals, limiting the growth of the crystals, and, therefore, the D50 of crystals was only 26.5 μm with the condition of 0.85 M NaCl in the solution. The microscopic images of the crystals also show the same trend, and the median crystal size was in the order of 0.75 M NaCl > 0.85 M NaCl > 0.65 M NaCl in the solution. D10 and D90 of crystals followed the same trend as D50 at 24 h. Crystals obtained in the condition of 0.75 M NaCl had the largest D10 of 11.5 μm and the largest D90 of 83.4 μm, and the crystals obtained in the condition of 0.85 M had the smallest D10 of 6.3 μm and the smallest D90 of 69.7 μm.

In the solutions with equal 0.65 M NaCl, the crystal yields increased with the increase in the lysozyme concentration, reaching 88% in the solution with 35 mg/mL lysozyme, as shown in Figure 5b. While the yield was about 40% in the solution mixed with 25 mg/mL lysozyme, there were no crystals obtained in the solution with 15 mg/mL lysozyme at 24 h. The crystals in the 35 mg/mL lysozyme experiment had the highest D50 of 23.7 μm in Figure 6b due to the faster nucleation and longer period for crystal growth. However, D90 of crystals obtained in the solution with 35 mg/mL lysozyme was smaller than those obtained with 25 mg/mL lysozyme.

The yields and crystal sizes were also compared when the concentration of protein in the solution became stabilized (slowly approaching solubility). One experiment was performed until reaching the equilibrium of the solution (concentration equals solubility) under in situ monitoring, and the time to reach equilibrium frequently took few weeks due to slow kinetics. Therefore, the experimental results of all experiments were only compared at the stabilized concentration stage. Figure 5a shows all experiments with 25 mg/mL lysozyme reached yields of more than 90%, indicating that the experiments were close to equilibrium at a stabilized stage. The highest yield of 99% was achieved in the solution of 0.85 M NaCl, and the lowest yield in the 0.65 M NaCl solution was 92%. There was an inverse relationship between the concentration of NaCl with the crystal sizes, and the crystals obtained in the solution with 0.65 M NaCl (D50 = 32.6 μm) > with 0.75 M NaCl (D50 = 30.6 μm) > with 0.85 M NaCl (D50 = 26.6 μm). D90 also followed the same trend as shown in Figure 6c. It is noted that low D10 in the experiment with 0.65 M NaCl and 25 mg/mL may be due to the formation of needle crystals in the solution (further discussed in the next section).

In the solutions with equal 0.65 M NaCl concentration, the crystal yields also increased with the increase in the lysozyme concentration, reaching 89% in the solution with 35 mg/mL lysozyme, as shown in Figure 5b. The increase of the key CSD parameters (D10, D50, and D90) had a similar trend to the one in increasing NaCl concentration. The median crystal size D50 was 55.5 μm with 15 mg/mL lysozyme, 32.6 μm with 25 mg/mL lysozyme, and 23.7 μm with 35 mg/mL lysozyme and that of D90 was 151.6 μm with 15 mg/mL lysozyme, 87.0 μm with 25 mg/mL lysozyme, and 70.3 μm with 35 mg/mL lysozyme. With similar supersaturation levels in the solution, the lower protein concentration resulted in larger average crystal sizes. For a supersaturation of about 5, the crystal obtained in the solution mixed with 25 mg/mL lysozyme, and 0.65 M NaCl was about 20% smaller than the crystals obtained in the solution mixed with 15 mg/mL lysozyme and 0.85 M NaCl.

**Figure 7:**
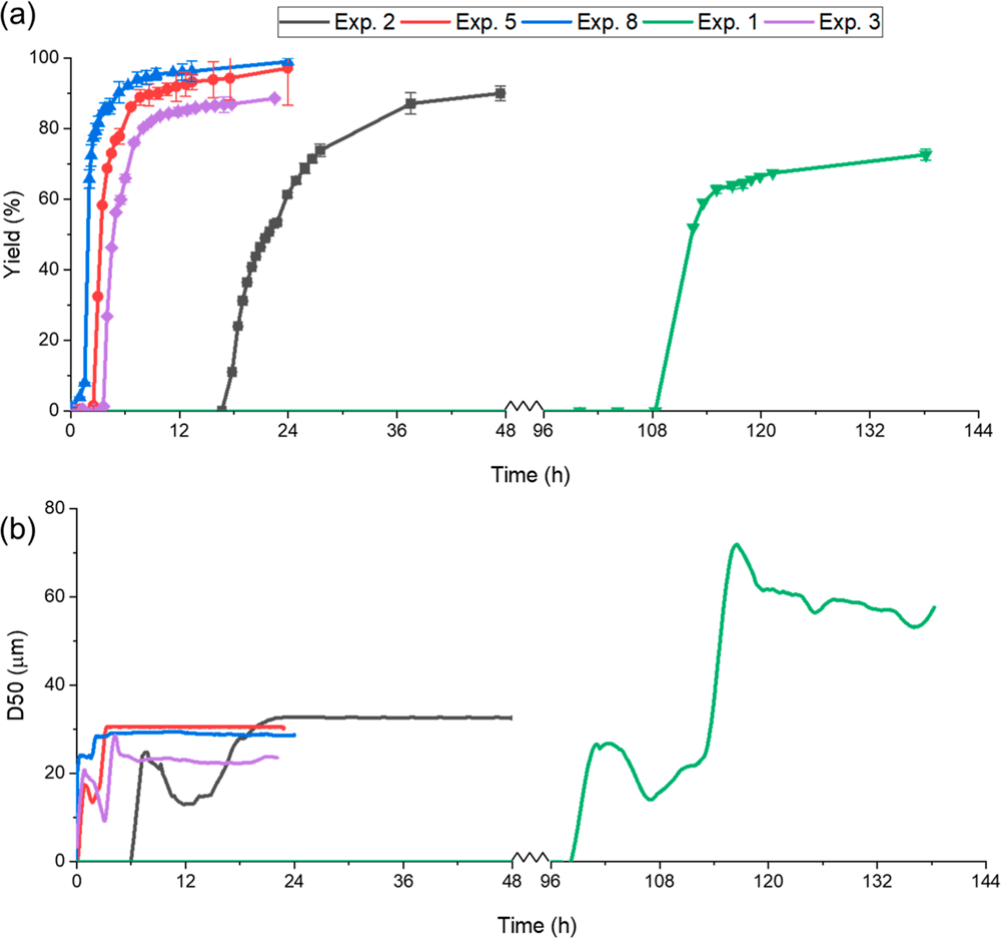
Crystal yield (a) and crystal median size D50 (b) in exps 1–3, 5, and 8 during the crystallization process.

Figure 7a shows that the maximum yields in these experiments were all over 70% but achieved at different time scales. In exp 8 with 0.85 M NaCl and 25 mg/mL lysozyme, the yield reached over 70% in the shortest period, followed by in exp 5, exp 3, exp 2, and in exp 1; the time for 70% yield was much longer than in other experiments, which was over 70 times longer than that in exp 2. The supersaturation was largest in exp 8 and lowest in exp 1, and a high supersaturation led to a fast nucleation, a fast rate of the concentration change, and a short time to achieve a high yield. The maximum yields for exp 2, exp 5, exp 8, and exp 3 were all over 80%, and the maximum yield for exp 2 was only about 70%. In all the experiments, the increase rates of the yields from above 0 to 90% were much faster than that from 90% to the maximum yield. For example, in exp 1, the yield was 0% until about 108 h. It took about 6 h from above 0% yield to 50% yield, but it took about 25 h from 50% yield to 70% yield (maximum yield achieved for exp 1). This trend was in agreement with stage 2 shown in Figure 4, when the high nucleation rate and rapid crystal growth occurred, and stage 3 when the system was approaching a stabilized state.

Figure 7b shows the that mean sizes of final crystal products were all over 20 μm in exp 3, about 30 μm in exp 2, exp 5, and exp 8, and over 60 μm in exp 1. The stabilized stage of the crystal size (the mean size remained in a similar range) was achieved at different time scales. The shortest time for achieving a stabilized stage in exp 6 was about 2.3 h, and the longest time was about 138.0 h in exp 1. The period for stabilizing D50 reduced with the increasing protein or NaCl concentration. This trend was consistent with the trend of the time to reach stage 3 in Figure 4. The largest D50 was observed in exp 1, which was more than twice larger than the D50 observed in all other conditions. The D50 of the crystals decreased with the increasing concentration of NaCl and lysozyme, shown in Figures 6 and 7. The trend was opposite to the trend of the supersaturation, except exp 7. As the supersaturation was determined by both NaCl and lysozyme concentrations, there was no linear correlation between CSD with supersaturation, similar to the corrections between induction time and supersaturations.

### Crystal Morphology and Bioavailability

**Figure 8:**
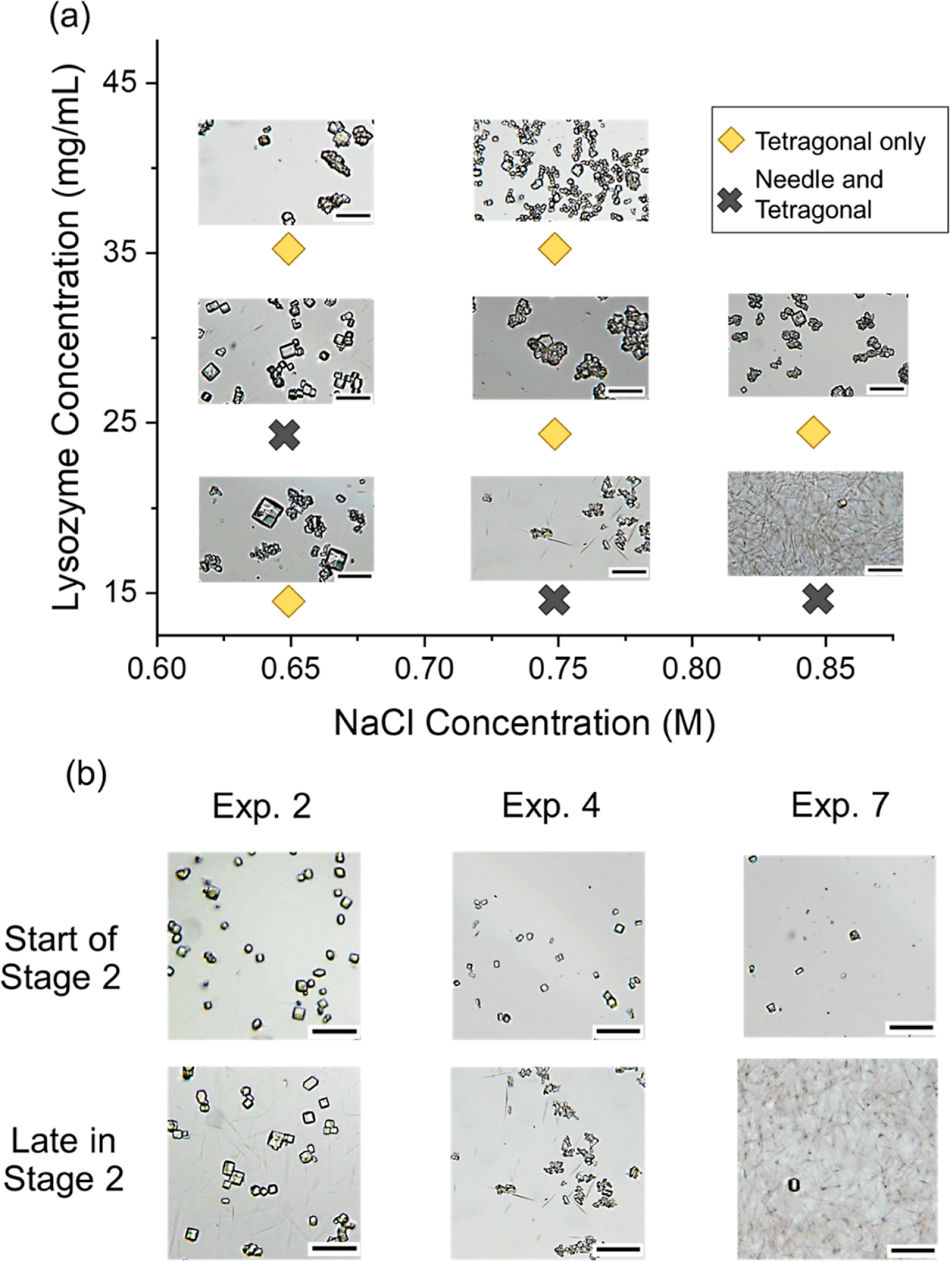
(a) Crystal with different morphologies obtained at stage 2 under different NaCl and protein concentrations. (b) Appearance of needle-like crystals during stage 2 in exps 2, 4, and 7. Scale bar: 150 μm.

The products obtained were mainly tetragonal-shaped crystals, and needle-shaped crystals appeared in exp 4, exp 7, and exp 2, as shown in Figure 8a. The needle-shaped crystals only appeared after the formation of tetragonal crystals in the solution. Figure 8b shows that in all the three experiments, there were only the tetragonal crystals at the start of stage 2. Later, the needle-shaped crystals were observed with larger tetragonal crystals. The appearance of the metastable form, the needle-shaped crystal in stage 2 and not in stage 1, was possibly a result of the secondary nucleation, which was consistent with the literature.^10^

As shown in Figure 8a, the number of needle-shaped crystals increased with the increase in the precipitant concentration with equal 15 mg/mL lysozyme. However, at 25 mg/mL lysozyme concentration, needle-shaped crystals were only produced in 0.65 M NaCl concentration, and no needle-shaped crystal was observed in the higher precipitant concentrations. It was reported that the formation of needle-shaped crystals was due to the secondary nucleation and low supersaturation,^10^ which was consistent with the phenomena in this work; the needle-shaped crystals only appeared in stage 2 with existing crystals in the solution. However, in the hanging drop experiments with very low supersaturation,^27^ there were no needle-shaped crystals observed under similar crystallization conditions. The shear was reported to influence the crystallization process and crystal morphology,^38,39^ and it was assumed that the shear also played an important role in the nucleation of the metastable needle-shaped crystal due to enhancement of mass transfer.

**Figure 9:**
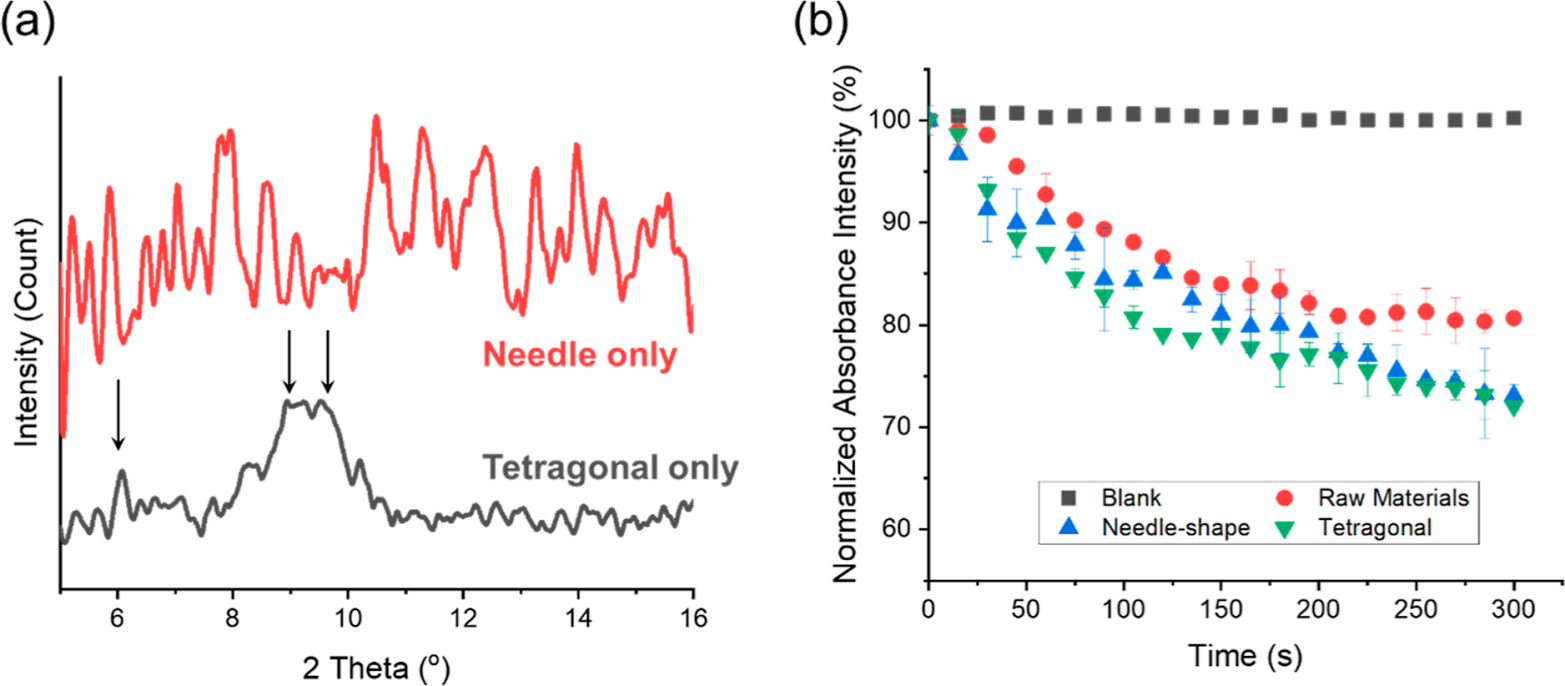
Product characterization of the needle-shaped and tetragonal crystals. (a) XRD patterns of two lysozyme crystal products with the needle-shaped crystal (by exp 7) and with tetragonal crystals (by exp 5). Arrows indicate the characteristics peak of at 6°, and between 9 and 10. (b) Normalized absorbance intensity at 450 nm of *Micrococcus lysodeikticus* cells without lysozyme (blank), with raw materials, with needle-shaped crystals (by exp 7), and with tetragonal crystals (by exp 5). The error bars represent the deviations in three repeated measurements.

Figure 9a shows the XRD patterns for the tetragonal and needle-shaped crystals obtained with different experimental conditions, and the diffraction data was collected from 5 to 16° to avoid the peaks of salt and buffer.^40,41^ Due to the complex structure of the protein, there were not many sharp peaks, but several different peaks indicate that the tetragonal and needle crystals had different crystalline structures. The XRD pattern of tetragonal crystals had peaks at 6°, and between 9 and 10°, which were in agreement with the literature.^40,41^

To analyze the bioactivity of the obtained crystal forms, the lysozyme activity was examined by the lysis process of *Micrococcus lysodeikticus* cells.^42^ The faster decrease of the absorption intensity means a higher speed of cell lysis, indicating higher activity. Figure 9b shows that both tetragonal crystals and needle-shaped crystals obtained in the experiments had about 40% higher bioavailability than the raw lysozyme materials (lyophilized powder) without regular shape. The order of bioavailability of these three different lysozyme forms is tetragonal crystals ≳ needle-shaped crystals > raw crystals. The total bioavailability of tetragonal crystals was slightly higher than that of the needle-shaped crystals, indicating that both needle-shaped crystals and tetragonal crystals were highly stable compared with raw materials, and the tetragonal crystals were slightly better than the needle-shape crystals in stability to preserve the more biological activity.

## Discussion

### Nucleation Rates

**Figure 10:**
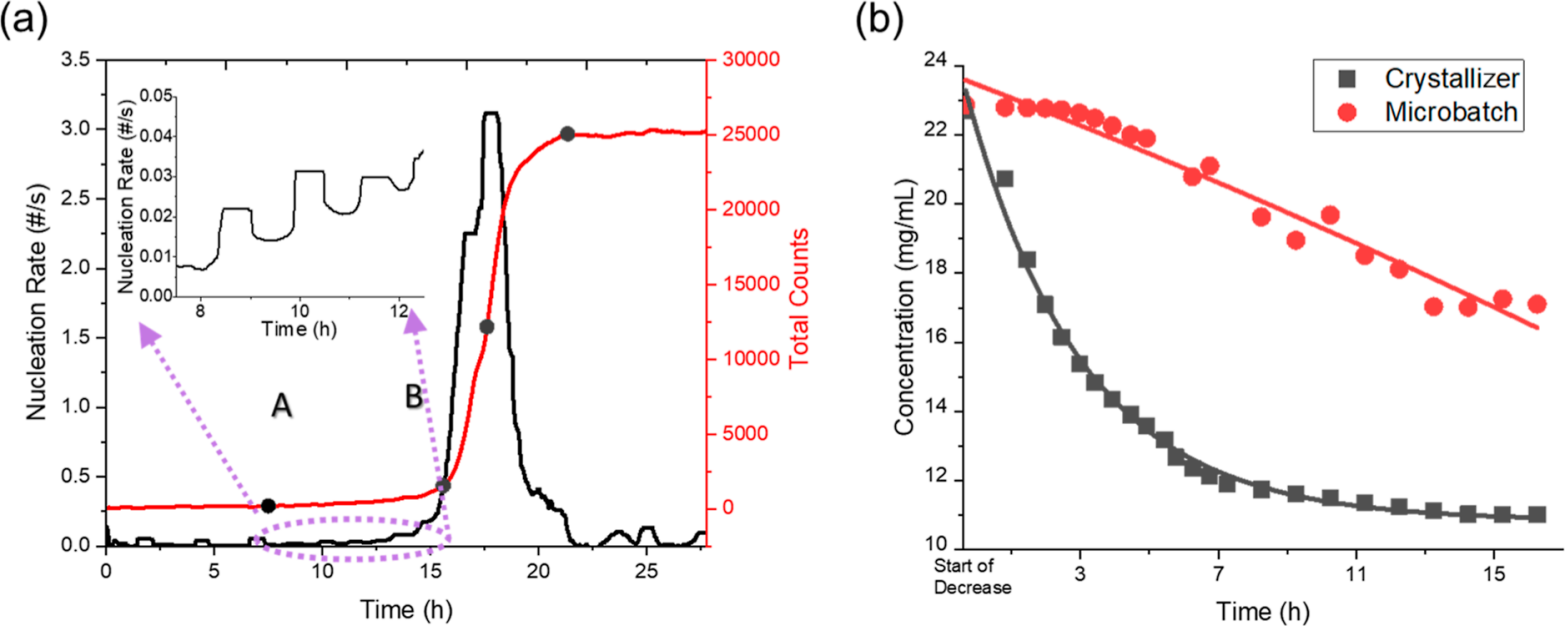
(a) Nucleation rates and total counts in exp 2. (b) Change of the lysozyme concentration after nucleation in exp 2 with stirring and microbatch without stirring in the solution with the equal crystallization condition as exp 2.

The nucleation mechanisms were different in stage 1 and stage 2 shown in Figure 3. Figure 10a shows the nucleation rates in exp 2, which were estimated based on the change of total counts over unit time. The nucleation rate starts to increase after point A (also marked in Figure 10), and the average nucleation rate was about 0.02 s^–1^ in stage 1. The average nucleation rate became 2.83 s^–1^ in stage 2, which was more than 100 times the nucleation rate in stage 1.

The significantly different nucleation rates in two stages should be due to the primary and secondary nucleation. After the formation of lysozyme crystals in stage 1, the stirring in the crystallizer easily broke lysozyme crystals by the contact with the stirrer and vessel walls,^43^ prompting the secondary nucleation in stage 2. With the same concentration of lysozyme and NaCl as in exp 2, the crystallization was performed without stirring in a microbatch of 5 mL. The starting point of decrease in the protein concentration in the crystallizer with stirring and in the microbatch without stirring was set as the initial point in Figure 10b. The decrease rate of the concentration in microbatch was much smaller than in exp 2, indicating that the stirring could accelerate the decrease of the lysozyme concentration due to the increase of the mass-transfer rate. Without stirring, the concentration decreased following a linear trend, and with stirring, the decrease rate of the concentration accelerated in the early stage, which agreed with the exponential growth reported in crystallization systems.^43^

However, it is still not fully understood that why the secondary nucleation started in a very late stage, which was hours after nucleation with thousands of crystals inside the solution. First, the protein crystals are always fragile,^2^ which should easily be broken inducing secondary nucleation. Second, the trigger of secondary nucleation was not clear. Assuming that there was a threshold of crystal numbers and crystal size to trigger secondary nucleation, the secondary nucleation should occur under stirring when there was a required amount of crystal numbers or crystals of sufficiently large size in the solution. The size distributions at the beginning of stage 2 for exps 1–9 were different, shown in Figure S3 in the Supporting Information, and, however, no good correlations were found between the size distribution (total counts and crystal size) to identify the threshold for secondary nucleation. One possible explanation is the formation of phase–phase separation as indicated in the two-step nucleation mechanism. During protein crystallization, protein-rich and protein-lean phases under supersaturated conditions were reported in many systems.^44−46^ The formation of dense liquid phase/droplets would be a metastable state before nucleation or storage of protein molecules for crystal growth.^47^ With dense phase droplets, the nucleation would be hindered as reported in other liquid–liquid-phase separation systems,^47,48^ and therefore, the nucleation rate in stage 1 was low. At stage 2, with the consumption of supersaturation, the liquid–liquid-phase separation would disappear, and crystals came out of the second-phase droplets tended to be easily broken by stirring. Nevertheless, over 100 times increase in the nucleation rate at stage 2 would also be still mainly contributed by secondary nucleation but potentially influenced by secondary-phase droplets. However, the mechanisms of nucleation rates and the needle-shaped crystals still need to be further investigated. Despite the need for further investigations, the thermodynamics and kinetics of the nucleation and crystal growth reported in his work provide a valuable foundation for simulating, scaling up, and designing the protein crystallization processes.

### Limitations of PAT

In this work, the concentrations were determined off-line due to the limitations of the PAT tools with the uncertainty and variations of on-line concentration measurements. Real-time monitoring of the crystallization process contributes to better understanding of nucleation and growth kinetics of pharmaceuticals, such as FBRM and PVM, in situ UV/vis spectrometry, and Raman spectroscopy.^48−50^ However, PVM cannot be efficiently applied in all experiments due to the formation and suspension of a large number of tiny crystals, especially the needle-like crystals in some experiments, and the images captured were difficult to distinguish single crystals (Figure S4 in the Supporting Information). The in situ concentration measurement in the crystallization solution was not possible because of the lack of characterization peaks and interference of crystal suspension in the solution.

An ATR-UV/vis spectrometer was tested based on the absorbance of lysozyme molecules in the UV/vis spectrum.^51^ The molecular weight of the lysozyme molecule is about 14 kDa with a huge number of bonds, leading to difficulty in identifying characterization peaks on the UV/vis spectrum (Figure S4 in the Supporting Information). The suspension of small crystals in the solution resulted in many obtrusions on the absorbance of lysozyme UV/vis spectrum for both in situ observations and the off-line measurements, which was in agreement with the literature that the tiny crystal in the solution would absorb the UV/vis spectrum.^52^ Several sample solutions from exps 1–6 were tested. The UV/vis spectrum absorbance of a sample solution with protein crystal suspension could be 3 times higher than the absorbance of the same solution after centrifugation (with the crystal suspension removed). In addition, the absorbance variation of the sample solution with crystal suspension was more than 150%, and the measurements of the sample solution after the centrifugation were consistent. In all of our measurements, the sample solutions were centrifugated first to avoid variations.

**Figure 11:**
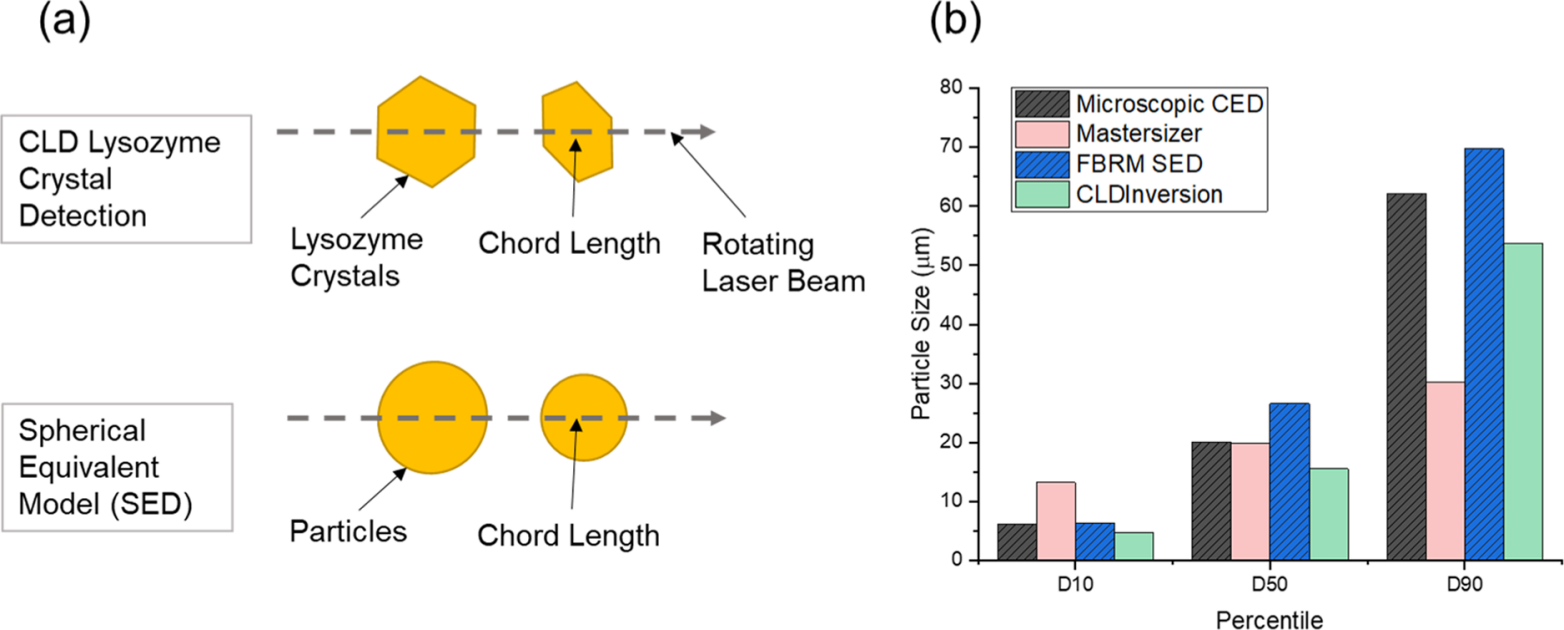
(a) Measurement of lysozyme chord length using FBRM and the spherical equivalent model estimation. (b) D10, D50, and D90 comparison of different CSD conversion methods for exp 8.

Four different methods were used to interpret the size distributions in this study. (i) FBRM–SED: SED model based on FBRM. The FBRM–SED model approximates the geometry of crystals based on the CLD of FBRM. A tetragonal lysozyme crystal was shown as an example for translating CLD to SED^16^ shown in Figure 11a. (ii) FBRM–CLD inversion: by applying comprehensive algorithm^17,18^ on the CLD of FBRM to search for the global best solution by finding the optimal aspect ratio for non-spherical crystals. (iii) Image circular equivalent model: by analyzing the microscopic images of the sample solution with crystals inside, the CSD can be estimated, but the frequency of the size distribution data will be limited by the sample interval. (iv) Laser diffraction from Mastersizer. Mastersizer provided a CSD, which requires a long time for preparation, such as filtration and drying, and also requires a certain amount of material. It is difficult to provide sufficient information for the whole process because it requires a high frequency of sampling and a high number of crystals in the sample solution (which is impossible for protein crystallization at a small scale). However, the CSD of the final crystal products can be used as the reference to compare with the other technologies.

Figure 11b shows D10, D50, and D90 estimated from methods for crystal products obtained in exp 8 at the end of the experiment. D10, D50, and D90 in size distribution from FBRM–CLD inversion were all lower compared to the one estimated by the image circular equivalent model. As CLD inversion utilized the database of small molecular crystallization, the difference in estimation indicated that the size distribution of protein crystals deviated from the small biopharmaceuticals.^17,18^ The CSD determined by the Mastersizer was in good agreement with the median size (D50) from the estimations from the image-circular equivalent model, however, with a larger value in D10 and a small value in D90. This is because the crystal sample measured by Mastersizer was filtrated. In addition, during the measurement of the Mastersizer, high stirring rates could result in the breakage of some agglomerations.

## Conclusions

With crystal images, size distribution, and concentration change during the protein crystallization process, the three stages in the protein crystallization process were identified as stage 1: slow nucleation (many tiny crystals observed without change in concentration), stage 2: rapid nucleation and growth (large number of crystals appeared and the size increased with fast decrease in concentration), and stage 3: slow growth and breakage (limited changes in crystal size and concentration). The nucleation rates in stage 2, which were hours later than the first occurrence of the nucleation, could be over 100 times higher than the nucleation rates in stage 1, when the concentration kept constant. The different nucleation rates were mainly due to the very slow kinetics and the secondary nucleation. The interfacial energy based on the nucleation in stage 1 was estimated to be 0.302–0.423 mJ/m^2^, with ln *A* to be 3.83–9.39, in the solution based on the equal NaCl concentrations of 0.65–0.85 M with different concentrations of lysozyme. The higher concentration of protein and NaCl led to higher yield, smaller average size, and higher possibility in the appearance of the needle-shaped crystals, with a different polymorph proved by XRPD. The needle-shaped crystals always appeared after the formation of the tetragonal crystals. The bioavailabilities of the tetragonal and needle-shaped crystals were both much higher than the raw materials, indicating that crystallization of protein can be a better method to keep high bioavailability than lyophilization.

## Supporting Information Available

Solubility of lysozyme, induction time estimated by different methods, crystal size distributions at different stages, limitation of the ATR-UV/vis spectrometer and PVM, and calibrations of the ATR-UV/vis spectrometer (PDF)
