# Supplementary material for: Protein Nucleation and Crystallization Process with Process Analytical Technologies in a Batch Crystallizer
Source: Cryst Growth Des. 2023 Jun 20;23(7):5181–93. doi: 10.1021/acs.cgd.3c00411 (PMC10326882; doi:10.1021/acs.cgd.3c00411)
Supplement: Supplementary file 1 — cg3c00411_si_001.pdf [file cg3c00411_si_001.pdf]

Protein Nucleation and Crystallisation Process with Process Analytical Technologies in a Batch Crystalliser

Wenqing Tian[a], Wei Li[a], and Huaiyu Yang[a*]

[a] *Department of Chemical Engineering, Loughborough University, Loughborough, LE113TU, UK*

SUPPORTING INFORMATION

Solubility of lysozyme

Figure S1 shows the solubility of tetragonal lysozyme crystals determined in this work, and the values were consistent with the values reported in the literature. The solubility determined in this work was used to calculate the supersaturation and product yield.

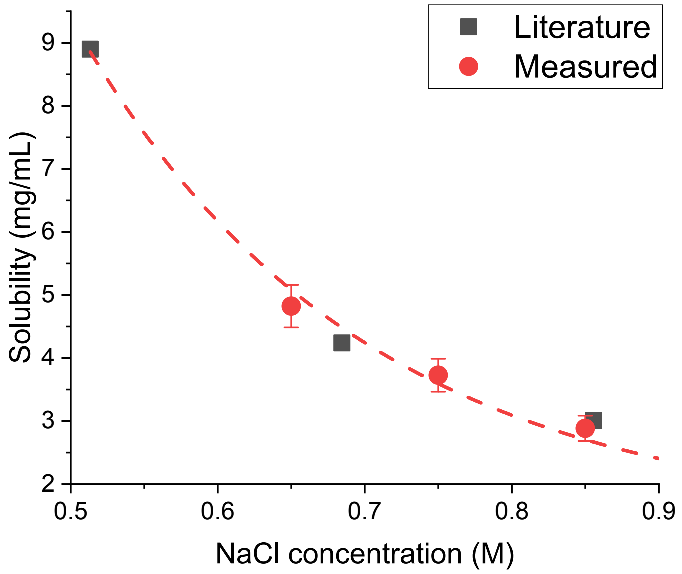

Figure S1. Solubility of tetragonal lysozyme crystals determined (circle) at 20°C in 0.1 M sodium acetate buffer with pH=4.2 and reported values[1] (square). The line is for guidance.

Induction time estimated by different methods

If the induction time was estimated by the starting of the decrease in lysozyme concentration, the measured induction time was always much longer than the induction time, detected by FBRM as the increase in the crystal count. As shown in Figure 3, the microscopic images of the process also proved that the lysozyme nuclei and crystal formation was before the decrease in the lysozyme concentration. The time for the concentration to drop can be up to 15 times longer than the appearance of tiny lysozyme crystals in the solution. The interfacial energy based on the concentration drop was estimated to be 0.488 mJ/m^2, 0.417 mJ/m^2, and 0.360 mJ/m^2, as shown in Figure S2, for 0.65 M, 0.75 M, and 0.85 M NaCl concentration respectively, which can be up to 20% differences compared to the interfacial energy estimated by the induction time determined with the FBRM curves. The pre-exponential factor, A, generally decreased with the increase of the NaCl concentration in the solution. Comparing the method of FBRM and concentration change, there was 95% difference in lnA in the solutions with 0.65 M NaCl, and the differences decreased with the increase of the NaCl concentration. In the solutions with 0.85 M NaCl, there was no obvious difference, possibly due to the significantly shortened nucleation time in in higher NaCl concentration.

Table S1. Interfacial energy, γ, and pre-exponential factor, A, estimated by the induction time based on method of FBRM and concentration change.

Methods	Nucleation parameter	Unit	NaCl concentration 0.65 M	0.75 M	0.85 M
FBRM	σ	mJ/m^2	0.42	0.39	0.30
	lnA	ln (s^{-1}•m^{-3})	9.39	4.81	3.83
Concentration change	σ	mJ/m^2	0.49	0.42	0.36
	lnA	ln (s^{-1}•m^{-3})	4.79	3.87	3.91

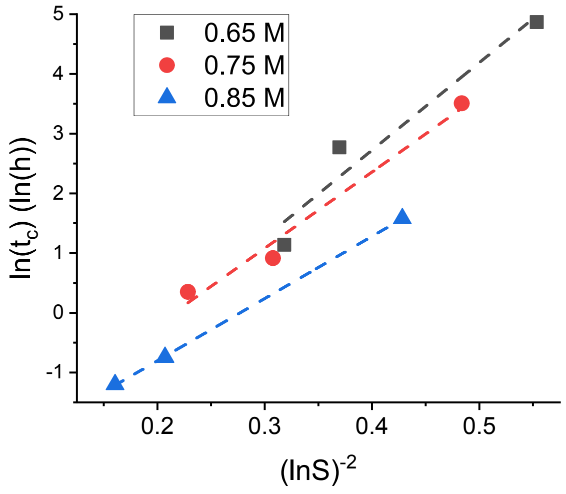

Figure S2. Correlations between S, and time, t_c, when the concentration started to drop at equal NaCl concentrations and different lysozyme concentrations. Dash lines are the best-fit linear lines for each NaCl concentration, respectively.

Crystal size distribution at different stages

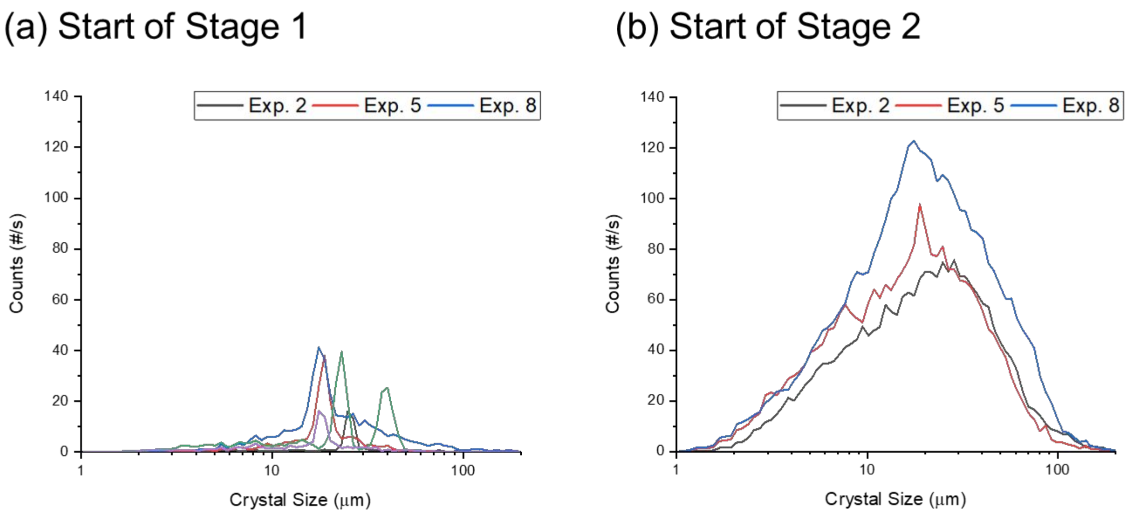

Figure S3. Cord length distributions at the beginning of Stage 1 (a) and 2 (b) in Exp. 2, 5 and 8.

The nucleation rate significantly changed between Stage 1 and Stage 2. The crystal size distributions at the beginning of Stage 1 and 2 of Exp. 2, 5, and 8 were compared in Figure S3 to find a threshold of crystal size distribution between Stage 1 and 2. Figure S3 (a) shows there were only limited particles at the beginning of Stage 1. After the slow nucleation period in Stage 1, there were much more crystal particles at the beginning of Stage 2. However, as shown in Figure S3 (b), the CLDs of the three experiments were not the same at the beginning of Stage 2, indicating there might be no strict threshold for the secondary nucleation to occur. The peak values of the distributions were in the same range and the total counts were in a similar range (1 or 2 order differences), suggesting a possible threshold range to trigger the secondary nucleation.

Limitation of ATR-UV/vis spectrometer and PVM

The recording of ATR-UV/vis spectrometer (Hellma ATR 661.822-UV probe) on Exp 3 is shown in Figure S4. Stepwise dilution of lysozyme solutions from a concentration of 70 mg/mL to 5 mg/mL was used to calibrate the correlation between absorbance at 280 nm and the lysozyme concentration. Three repeated experiments were carried out to find the correlation. To eliminate the background noise, the absorbance was recalibrated with the lysozyme concentration at beginning of each crystallisation experiment.

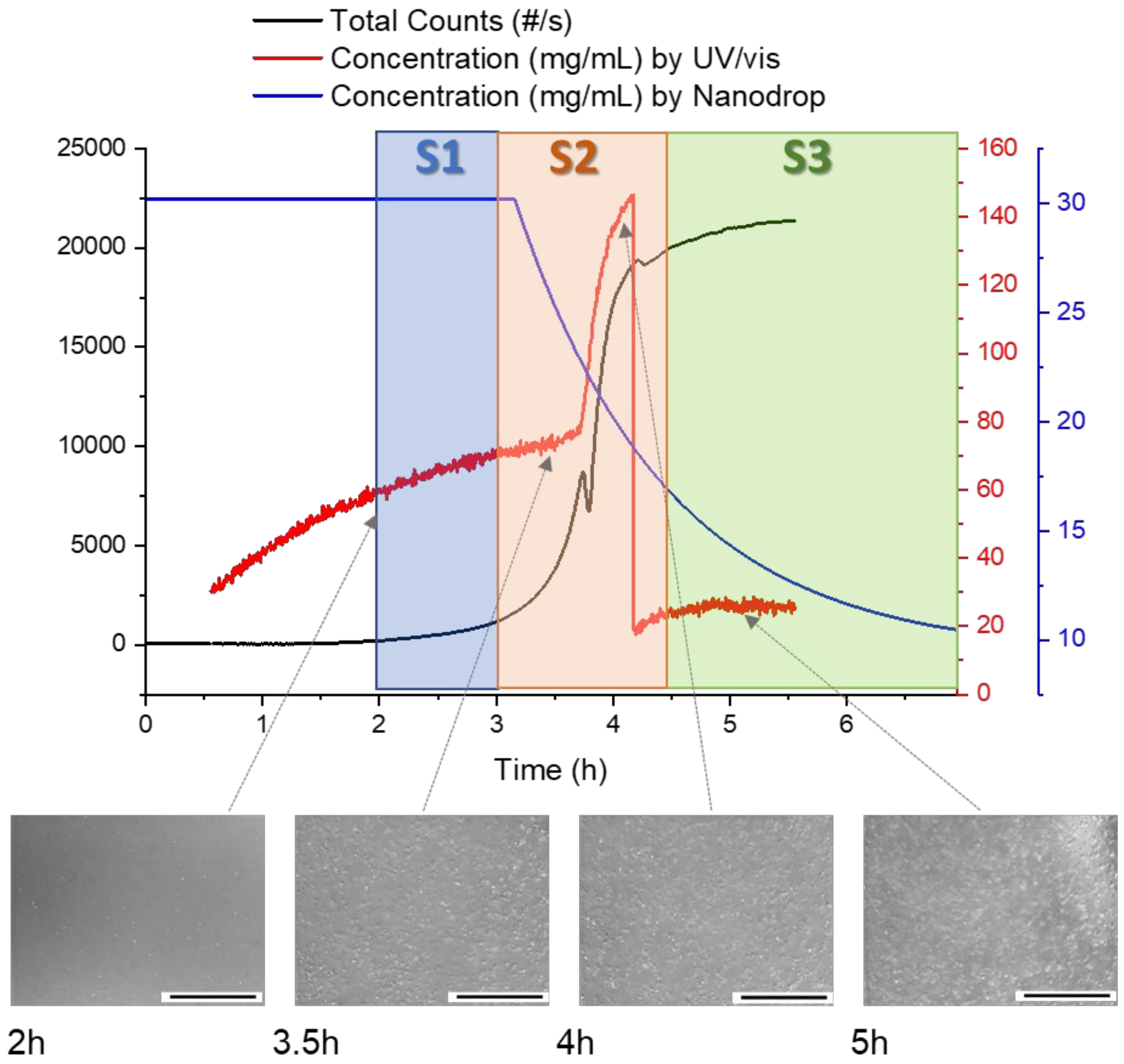

Figure S4. Total counts evolution, concentration changes based on in-situ UV/vis spectrometer and ex-situ Nanodrop spectrophotometer, and PVM images in Exp .3. Scale bar: 200 μm.

The absorbance intensity in Exp.3 continually increased in Stage 1, the slow nucleation stage. One possible reason for the increase of the absorbance intensity in Stage 1 was due to the tiny solid lysozyme or the aggregated lysozyme particles in the solution, i.e. in

the near front of the spectrometer probe, which was consistent with the off-line spectrometer observation that without filtration the concentration reading was influenced by the tiny crystals in the droplet solution. The absorbance intensity had a significant jump at middle of Stage 2, and at the same period FBRM show a dive and jump patten, which was similar to the nucleation in small moecules due to heat of the nucleation. However, the jump of absorbance was not fully understood, may be due to the phase change or temperature change. After short period, the absorbance intensity of had a steep fall close to the end of Stage 2, maybe due to the growth of the crystals, there was limited influence of the tiny crystals on the probe. Therefore, the absorbance intensity decreased to very low level which was reasonable due to the concentration of the solution highly decreased after nucleation and crystal period. The absorbance intensity keep consistent after 4.5 hour when entering into Stage 3. It is noted that the phenomena of the absorbance intensity during the whole process were similar to the nucleation and crystal growth process of small molecules in liquid-liquid phase separation solution [2]. In sum, the complex solution environment influence the absorbance intensity with *in-situ* UV/vis spectroscopy, and, therefore, absorbance intensity was not reliable to directly correlated to protein concentrations during the protein crystallisation process, i.e. the protein concentration in the solution was hardly estimated from UV/vis absorbance intensity.

PVM images during the crystallisation process were obtained, shown in Figure S4. The PVM probe was obstructed by fouling, as a large amount of tiny protein crystals formed on the PVM probe window. There was limited information that can be interpreted due to the fouling, but the microscopic analysis with the sample from the solution could provide

more detailed information, such as crystal shape and size distributions during the protein crystallisation process.

Calibrations of ATR-UV/vis spectrometer

According to the Beer-Lambert law, absorbance and lysozyme concentration have a linear relationship. Characteristic wavelength was chosen based on the analysing the absorbance spectrum of the lysozyme solution and the largest change in absorbance when changing lysozyme concentration was selected.

The calibration of ATR-UV/vis spectrometer was carried out by the step-wise dilution of lysozyme solution with known concentrations. The concentrations were verified by using offline Nanodrop© spectrophotometer. The correlation of lysozyme concentration and absorbance of characterisitic wavelength is shown in Figure S5. The linear regression coefficient was found to be 0.99, which indicated a linear correlation as described before.

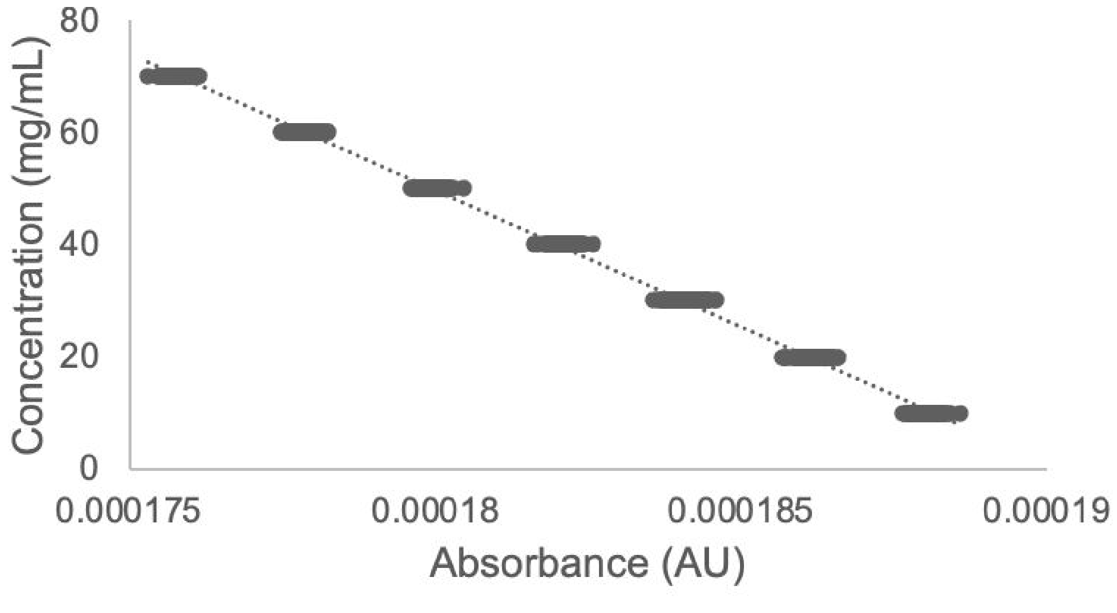

Figure S5. Calibration of absorbance and lysozyme concentration. Dashing line is the linear regression of concentration and absorbance.

REFERENCE

(1) Forsythe, E. L.; Judge, R. A.; Pusey, M. L. Tetragonal Chicken Egg White Lysozyme Solubility in Sodium Chloride Solutions. *J Chem Eng Data* **1999**, *44* (3), 637–640. https://doi.org/10.1021/je980316a.

(2) Wang, L.; Bao, Y.; Sun, Z.; Pinfield, V. J.; Yin, Q.; Yang, H. Investigation of Agglomeration in the Presence of Oiling out in the Antisolvent Crystallization Process. *Ind Eng Chem Res* **2021**, *60* (10), 4110–4119. https://doi.org/10.1021/ACS.IECR.1C00491/SUPPL_FILE/IE1C00491_SI_001.PDF.
